# Clinical Picture and Risk Factors for Severity of SARS-CoV-2 and Dengue Coinfection in Children: Experience From a Tertiary Hospital in Vietnam

**DOI:** 10.7759/cureus.66535

**Published:** 2024-08-09

**Authors:** Nguyen The Nguyen Phung, Minh Nhut Tran, Thanh Thuc Tran, Duy Minh Vo

**Affiliations:** 1 Pediatrics, University of Medicine and Pharmacy at Ho Chi Minh City, Ho Chi Minh City, VNM; 2 Infectious Diseases Intensive Care Unit, Children Hospital No. 1, Ho Chi Minh City, VNM

**Keywords:** risk factor, children, vietnam, pediatric, coinfection, sars-cov-2, covid-19, dengue

## Abstract

Introduction

Dengue is an infectious disease that is a burden in Asia-Pacific and Latin America. The COVID-19 pandemic in dengue-endemic areas has caused a "double burden" because of the possibility of coinfection, especially in children who are vulnerable to both COVID-19 and dengue. This study aimed to describe the characteristics and identify risk factors for the severity of the coinfection in Vietnamese children.

Methods

This was a retrospective cohort study, undertaken at Children's Hospital 1 (Ho Chi Minh City, Vietnam) during the fourth wave of the COVID-19 pandemic. All children under 16 years old who were admitted to the hospital from April 27, 2021 to June 30, 2022, and diagnosed with SARS-CoV-2 and dengue coinfection were included.

Results

From April 2021 to June 2022, a total of 31 patients with the coinfection were included, with 19 of them being male (61.3%). The median age was 10.8 years old (IQR, 5.1-14.1). Fourteen children (45.2%) had preexisting comorbidities, with the most common comorbidity being overweight/obesity (ten children). Nearly two-thirds of the children were diagnosed with dengue without/with warning signs (61.3%) and were classified as having mild COVID-19 (83.9%). The most frequently observed clinical characteristics were fever (n=29, 93.6%), followed by abdominal pain, vomiting, and petechiae. All patients had high serum ferritin, and 83.9% presented with thrombocytopenia. None of the cases died. Overweight/obesity, abdominal pain, and petechiae were factors independently associated with severe disease.

Conclusion

Most of the children had mild COVID-19 and disease progression similar to patients with dengue alone. However, some children may have severe COVID-19 and dengue coinfection. Obesity, abdominal pain, and petechiae were identified as independent risk factors for disease severity in pediatric cases. Further studies with multicenters and a larger sample size are needed to assess the coinfection more thoroughly.

## Introduction

Dengue is caused by the dengue virus (DENV) and spreads from its vectors (Aedes aegypti and Aedes albopictus mosquitoes) to human beings [1]. Dengue is more common in tropical and subtropical climates, causing epidemics in the Asia-Pacific and Latin America regions [1,2]. Over the past decade (2010-2020), it is estimated that there were 390 million cases of DENV infection overall, including 96 million symptomatic cases, and 2 million cases of severe illness, with 21,000 deaths worldwide per year [1]. In Vietnam, a country located in the endemic area of DENV, dengue outbreaks occur mainly during the monsoon (June to October), mostly in the southern provinces; however, dengue cases can appear all year round [2-4]. The highest incidence of DENV infection occurs in Asia, where children between 5 and 15 years of age are primarily affected [1].

The COVID-19, caused by SARS-CoV-2, originated in December 2019 and then rapidly spread to more than 200 countries, including Vietnam [5,6]. The spread of SARS-CoV-2 in dengue-endemic areas has become a 'double burden' for many countries in the Pacific-Asia region because of the similarities in clinical and laboratory findings of these diseases, which are sometimes difficult to distinguish and decide to treat accurately, as the two diseases are managed differently [3,5].

Children are vulnerable to SARS-CoV-2 infection because immunization in children is not widely available, and they are also susceptible to DENV. However, there are few studies about SARS-CoV-2 and dengue coinfection in pediatrics, as most studies have been reported in adults [3,5,7]. Current literature reports many cases of coinfection, in which results show a higher morbidity and mortality in coinfection cases [8]. During the fourth wave of the COVID-19 pandemic in Vietnam (starting in April 2021), while the predominant SARS-CoV-2 variants were Delta and Omicron, many cases of coinfection have been recorded [9-12]. Understanding the pathogenesis, management, and prognosis of the coinfection remains limited. Some studies have shown that in adults, patients with comorbidities, especially obesity, diabetes, and cardiovascular disease, had a significant risk for severe disease and death [5,8]. This study aimed to describe the clinical and laboratory features, diagnosis, and management of COVID-19 and dengue coinfection in Vietnamese children admitted to a tertiary pediatric hospital in Ho Chi Minh City, Vietnam; additionally, we also present risk factors for disease severity in these patients.

## Materials and methods

Study population

We retrospectively assessed all children diagnosed with SARS-CoV-2 and dengue coinfection during the fourth wave of COVID-19 (from April 27, 2021 to June 30, 2022) admitted to Children's Hospital 1 in Ho Chi Minh City, Vietnam. Children’s Hospital 1 is a tertiary hospital for children (0-16 years old) in the southern region of Vietnam.

The diagnosis and management strategies for dengue and COVID-19 cases were based on the guidelines of the Ministry of Health (Vietnam) [4,9]. Severity classification of DENV infection includes dengue without warning signs, dengue with warning signs, and severe dengue hemorrhagic fever (Appendix 1) [4]. The severity of COVID-19 can be classified as mild, moderate, severe, or critical (Appendix 2) [9].

Inclusion criteria

The study enrolled children under 16 years of age who were diagnosed with SARS-CoV-2 and dengue coinfection if they met the following three criteria: (1) Symptoms and signs within 15 days; (2) Patients had a fever ≥ 38°C or possibly other symptoms such as nausea, vomiting, abdominal pain, or signs of bleeding; (3) Patients had a positive real-time RT-PCR for SARS-CoV-2 from nasopharyngeal swab specimens, and either detection of non-structural protein 1 antigen (NS1Ag) (for patients with less than 5 days from illness onset) or positive serological tests detecting IgM via enzyme-linked immunosorbent assay (ELISA) (for patients with five days or more from illness onset).

Data collection

Information on demographics, clinical and laboratory features, comorbidities, diagnosis, management, and outcomes was recorded for all patients who met the inclusion criteria by reviewing their medical records and documenting it in a unique medical report form. All patients were routinely tested with a complete blood count. Other laboratory tests, including C-reactive protein (CRP), ferritin, liver function, kidney function, complete coagulation status, and chest X-ray, were only taken in moderate/severe/critical COVID-19 cases or cases diagnosed with dengue with warning signs/severe dengue hemorrhagic fever. The NS1Ag test used at our hospital was the Dengue NS1 Antigen Test Kit (Humasis Company, South Korea) and the Dengue IgM ELISA test was the NovaLisa Test Kit (NovaTec Company, Germany). SARS-CoV-2 RT-PCR was performed on CFX96 Touch Real-Time PCR Detection System (BIO-RAD) and Realtime PCR Rotor-Gene Q MDx (QIAGEN) at the Department of Microbiology of the hospital (licensed by the Ministry of Health of Vietnam). The target genetic region for SARS-CoV-2 Real-time RT-PCR test was the E gene.

The nutritional status of the children was classified based on WHO criteria, including: Patients 0-5 years old: Weight-for-height (WH) < -2SD: Malnutrition; -2SD ≤ WH ≤ 2SD: Normal; WH > 2SD: Overweight/Obesity. Patients more than 5-16 years old: BMI < -2SD: Malnutrition; -2SD ≤ BMI ≤ 1SD: Normal; BMI > 1SD: Overweight/Obesity. We divided all participants with SARS-CoV-2 and dengue coinfection into two groups: severe (severe dengue hemorrhagic fever and/or severe/critical COVID-19) and non-severe (dengue without/with warning signs and mild/moderate COVID-19).

Statistical analysis

STATA software version 14.2 (STATA Corp LLC, 4905 Lakeway Drive, College Station, TX, USA) was used for statistical analysis. Results were presented as frequencies (n) and percentages (%) for categorical variables. For continuous variables, results were expressed as the mean ± SD if the distribution of data was regular, and as the median and IQR if the data were not normally distributed. If a laboratory test had multiple results, we took the largest value among those results. Differences between the severe and non-severe groups were analyzed using the Chi-square test or Fisher's exact test for categorical variables, and the Mann-Whitney U test was used to compare the median values for non-normally distributed data. The results were considered statistically significant when the p-value was less than 0.05, with a 95% CI. Multivariable logistic regression analysis was used to identify independent factors associated with severe disease.

Ethical approval

This study was approved by the Medical Ethics Committee of Children's Hospital 1, with the research code CN/N1/21/65.

## Results

From April 2021 to June 2022, a total of 31 patients were included in the study, with 19 of them being male (61.3%). The median age in our study was 10.8 years old (IQR, 5.1-14.1), with the youngest patient being an eight-month-old child and the oldest being 15.5 years old. Eighteen children (58%) were 12 years old or older. In the early stage of the pandemic (before October 2021), the coinfection was diagnosed in only four children (12.9%). After lockdowns were eased (from October 2021), more children were infected (87.1%).

Fourteen children (45.2%) had pre-existing comorbidities. The most common comorbidity was overweight/obesity (n = 10, 32.3%), with seven children belonging to the severe group (n = 7/12, 58.3%) and three children having moderate to severe/critical COVID-19 (n = 3/5, 60%). Other underlying diseases included leukoencephalopathy with developmental delay (n = 1), asthma (n = 1), renal cysts (n = 1), and chronic thrombocytopenic purpura (n = 1). No children were malnourished. None of the children had been immunized with the COVID-19 vaccine.

Most children belonged to the non-severe group (n = 19, 61.3%), and the majority had mild COVID-19 (n = 26, 83.9%). Dengue shock syndrome (DSS) usually occurred on the fifth day from illness onset. Details about dengue and COVID-19 severity are shown in Table 1.

**Table 1:** Dengue and COVID-19 severity classification in children with the coinfection. * Severe cases with the coinfection.

		COVID-19 Classification		
		Mild (n = 26)	Moderate (n = 3)	Severe/Critical (n = 2)
Dengue Classification	Without warning signs (n = 12)	11	1	0*
	With warning signs (n = 7)	7	0	0*
	Severe (dengue hemorrhagic shock, organ failure) (n = 12)	8*	2*	2*

Fever was the most common chief complaint for hospitalization (90.3%), followed by vomiting (6.4%) and seizures (3.3%). The average time from illness onset to hospitalization was 4.2 ± 1.6 days. Demographic characteristics and clinical manifestations were presented in Table 2. The most common clinical feature was fever with a median duration of 5 days; followed by vomiting, abdominal pain, and petechiae. DSS was seen in 12 patients (38.7%). Bleeding complications occurred in fourteen patients, including nine with DSS. All patients were diagnosed with DENV infection at the time of admission, 87% based on positive NS1Ag and 13% based on positive Dengue IgM ELISA.

**Table 2:** Demographics and clinical manifestations in children with the coinfection: comparisons between severe and non-severe groups. * The bold formatting is signaling statistical significance. ^a^ Chi-square test;  ^b^ Fisher’s exact test.

Characteristics	Total	Severity of the coinfection		P-value
	(N = 31)	Severe (N = 12) n (%)	Non-severe (N = 19) n (%)	
Demographics				
Sex				1.000^b^
Male	19 (61.3)	7 (58.3)	12 (63.2)	
Female	12 (38.7)	5 (41.7)	7 (36.8)	
Age (years old)				
<5	7 (22.6)	3 (25.0)	4 (21.1)	1.000^b^
6-11	6 (19.3)	2 (16.7)	4 (21.1)	1.000^b^
≥12	18 (58.1)	7 (58.3)	11 (57.8)	0.981^a^
Overweight/Obesity	10 (32.3)	7 (58.3)	3 (15.8)	0.021^b^
Clinical manifestations				
Fever	29 (93.5)	11 (91.7)	18 (94.7)	1.000^b^
Vomiting	13 (41.9)	6 (50.0)	7 (36.8)	0.470^a^
Abdominal pain	13 (41.9)	9 (75.0)	4 (21.1)	0.003^a^
Petechiae	11 (35.5)	8 (66.7)	3 (15.8)	0.007^b^
Mucosal bleeding	3 (9.7)	3 (25.0)	0 (0.0)	0.049^b^
Rash	5 (16.1)	2 (16.7)	3 (15.8)	1.000^b^
Cough	9 (29.0)	4 (33.3)	5 (26.3)	0.704^b^
Dyspnea/Tachypnea	6 (19.4)	6 (50.0)	0 (0.0)	0.001^b^
Diarrhea	4 (12.9)	1 (8.3)	3 (15.8)	1.000^b^
Loss of taste/smell	3 (9.7)	1 (8.3)	2 (10.5)	1.000^b^
Sore throat	4 (12.9)	1 (8.3)	3 (15.8)	1.000^b^
Arthralgia	1 (3.2)	0 (0.0)	1 (5.3)	1.000^b^
COVID-19 severity				0.082^b^
Mild	26 (83.9)	8 (66.6)	18 (94.7)	0.060^b^
Moderate	3 (9.7)	2 (16.7)	1 (5.3)	0.543^b^
Severe/Critical	2 (6.5)	2 (16.7)	0 (0.0)	0.142^b^

Laboratory findings of patients with the coinfection in general and in two groups are presented in Table 3. One patient had an AST level of more than 1000 U/L. Elevated CRP greater than 20 mg/L was identified in five patients (20.8%). All patients had increased levels of serum ferritin. The most common laboratory features were high ferritin, thrombocytopenia, and normal CRP. In two patients with both severe dengue and severe COVID-19, common laboratory findings included lymphopenia, elevated liver enzymes, high serum ferritin, thrombocytopenia, and pleural effusion on chest X-ray. Chest radiographs were indicated in six patients, with the most common features being pleural effusion (four patients), interstitial lesions (two patients), parenchymal consolidation (two patients), and atelectasis (one patient).

**Table 3:** Laboratory features in children with the coinfection: comparison between severe and non-severe groups. *The bold formatting is signaling statistical significance. ^a^ Chi-square test;  ^b^ Fisher’s exact test;  ^c^ Mann-Whitney U test NEU: Neutrophil count; LYM: Lymphocyte count; PLT: Platelet count; PT: Prothrombin time; aPTT: Activated partial thromboplastin time; CRP: C-reactive protein; AST: Aspartate aminotransaminase; ALT: Alanine aminotransaminase; Ct: Cycle threshold; N: Number of cases; NA: Not available. Reference range: WBC: 4.0-12.0 (x10^3^/µL); NEU: 1.5-8.5 (x10^3^/µL); LYM 1.5-3.0 (x10^3^/µL); Hb: 11.5-14.5 (g/dL); PLT: 150-400 (x10^3^/µL); PT: 13.1-16.3 (seconds); aPTT: 28.6-35.8 (seconds); Fibrinogen: 2.03-3.77 (g/L); D-Dimer: 0.09-0.53 (mg/L); CRP: < 5 (mg/L); Ferritin: 6-60 (µg/L); AST: 15-60 (U/L); ALT: 13-45 (U/L); Creatinine: 35.0-62.0 (µmol/L).

Parameters (units)	Total		Severity of the coinfection				P-value
			Severe (n = 12)		Non-severe (n = 19)		
	N	n (%) or Median (IQR)	N	n (%) or Median (IQR)	N	n (%) or Median (IQR)	
WBC (x103/µL)	31	5.1 (3.3-6.9)	12	5.9 (3.0-7.5)	19	5.03 (3.3-6.8)	0.478c
Leukocytosis		2 (6.5)		2 (16.7)		0 (0.0)	0.142b
Leukopenia		14 (45.2)		5 (41.7)		9 (47.4)	0.756a
NEU (x103/µL)		1.92 (1.26-3.77)		2.36 (1.16-5.44)		1.86 (1.48-2.96)	0.570c
Neutropenia		10 (32.3)		4 (33.3)		6 (31.6)	1.000b
LYM (x103/µL)		1.61 (1.11-3.44)		1.82 (0.78-3.60)		1.59 (1.13-2.73)	0.887c
Lymphopenia		16 (51.6)		6 (50.0)		10 (52.6)	0.886a
Hemoglobin (g/dL)		13.7 (13.1-15.4)		13.8 (13.1-16.4)		13.7 (12.2-15.2)	0.522c
PLT (x103/µL)		70 (26-131)		30 (21.5-80)		101 (41-136)	0.045c
Thrombocytopenia		26 (83.9)		11 (91.7)		15 (79.0)	0.624b
PT (seconds)	14	14.8 (13.7-16.4)	8	14.8 (14.0-18.1)	6	14.8 (13.6-16.1)	0.698c
Prolonged PT		2 (14.3)		2 (16.7)		0 (0.0)	0.473b
aPTT (seconds)		39.2 (35.4-41.9)		40.6 (36.6-45.2)		38.2 (30.8-40.0)	0.245c
Prolonged aPTT		2 (14.3)		2 (16.7)		0 (0.0)	0.473b
Fibrinogen (g/L)		2.08 (1.62-2.32)		1.84 (1.48-2.22)		2.22 (2.03-2.50)	0.156c
D-dimer (mg/L)	12	1.57 (0.53-2.67)	7	1.35 (0.59-2.40)	5	2.16 (0.42-3.67)	0.807c
Elevated D-dimer		3 (25.0)		1 (8.3)		2 (10.5)	0.523b
CRP (mg/L)	24	3.47 (2.30-13.20)	10	4.18 (2.84-7.23)	14	3.16 (1.95-20.50)	0.953c
CRP > 20mg/L		5 (20.8)		1 (8.3)		4 (21.1)	0.358b
Ferritin (µg/L)	23	2344.9 (707.3-5311.3)	9	3164.8 (1669.7-5311.3)	14	1825.9 (510-5265.5)	0.231c
Elevated Ferritin		23 (100.0)		9 (100.0)		14 (100.0)	NA
AST (U/L)	8	217.0 (145.5-581.0)	6	329 (139.8-690.8)	2	199.2 (151.2-247.3)	0.739c
Elevated AST		8 (100.0)		6 (100.0)		2 (100.0)	NA
ALT (U/L)		159.9 (59.6-293.4)		159.9 (69.5-352.3)		142.1 (49.7-234.4)	0.739c
Elevated ALT		7 (87.5)		6 (100.0)		1 (50.0)	0.250b
Creatinine (µmol/L)	8	64.2 (46.7-73.6)	6	64.2 (49.9-69.4)	2	60.7 (43.5-77.8)	1.000c
E gene Ct value	31	29.6 (20.0-35.8)	12	34.6 (24.3-36.4)	19	28 (19.7-32.1)	0.273c

When comparing two groups (severe and non-severe), we identified risk factors for disease severity, including overweight/obesity, abdominal pain, petechiae, mucosal bleeding, dyspnea/tachypnea, and platelet count. We used multivariable logistic regression analysis to find independent factors associated with severe disease. Overweight/obesity, abdominal pain, and petechiae were factors independently associated with severe disease (Table 4). The median platelet count was lower (less than 100x10^3^/µL) in the severe group compared to the non-severe group, and the difference was statistically significant. The number of patients with mild COVID-19 in the non-severe group was higher than in the severe group (94.7% versus 66.6%), but the difference was not statistically significant.

**Table 4:** Multivariable logistic regression analysis of independent factors between severe and non-severe group. *The bold formatting is signaling statistical significance. NA: Not available

Characteristics	Odd ratio	P-value	95% CI
Overweight/obesity	7.47	0.014	1.086-58.466
Abdominal pain	11.25	0.003	1.610-89.614
Petechiae	10.667	0.004	1.497-86.043
Mucosal bleeding	NA	NA	NA
Dyspnea/Tachypnea	NA	NA	NA

Antibiotics were used as part of the management in five children (16.1%) because bacterial coinfection in these children could sometimes not be ruled out. Antibiotics used in our children included third-generation cephalosporins (three children); a combination of carbapenem and vancomycin (two children). The rate of antibiotic use in the non-severe dengue group was 15.8% (n = 3) and in the severe group was 16.7% (n = 2). The antiviral drug Remdesivir was used in one patient; dexamethasone was used in three children, including two with severe/critical COVID-19 and one with moderate COVID-19. Low molecular weight heparin (LMWH) was used in two children at a dose of 1 mg/kg/day (one child with severe/critical COVID-19 and severe dengue) and 2 mg/kg/day (one child with moderate COVID-19 and dengue without warning signs). Twelve children with DSS were given fluid therapy according to clinical guidelines. Five children received respiratory support, including oxygen (two), continuous positive airway pressure (CPAP) (two), and mechanical ventilation (one). The patient receiving mechanical ventilation was diagnosed with severe COVID-19 and severe dengue. This patient was given several antibiotics including carbapenem, vancomycin; Remdesivir, LMWH, and dexamethasone. All patients had a good recovery. The average hospital stay was 5.77 ± 3 days, with the longest hospital stay being 14 days.

## Discussion

In the context of SARS-CoV-2 still progressing and new variants appearing, children can be coinfected with SARS-CoV-2 and dengue, and therefore, the coinfection could still become a “double burden” for the health system in the future [13,14]. During the fourth wave of COVID-19, a lockdown was imposed in Ho Chi Minh City, which reduced the number of diagnosed dengue cases. When the lockdown was lifted (October 2021), and the vaccine was available to people over 18 years old, we observed an increase in the number of children with coinfection. Both dengue and COVID-19 did not have suitable vaccines for children at the time the patients in our study were hospitalized. Currently, according to the latest guidelines of the Ministry of Health (Vietnam), COVID-19 vaccination with Pfizer-BioNTech vaccines is carried out for children aged 6 months and older [9,10].

COVID-19 and DENV infection have similar pathogenesis, so both diseases show significant similarities in clinical and subclinical manifestations [3,5]. Some children exhibit symptoms such as skin and mucosal bleeding, shock, and retroorbital pain, which are more indicative of DENV infection than COVID-19. In contrast, symptoms such as dry cough, sore throat, tachypnea/dyspnea, and loss of taste/smell are common in COVID-19 [3,15]. Determining coinfections is crucial for monitoring, management, preventing deterioration, and effectively isolating and controlling infections. In our study, more than 90 percent of all children had fever, followed by vomiting and abdominal pain in nearly half of the cases. These symptoms are common in any viral infection [3,15,16]. During the COVID-19 pandemic, detection of coinfection became easier because screening for SARS-CoV-2 was performed routinely. The problem now is that when the COVID-19 pandemic has passed, missing the diagnosis of coinfection may occur, so if a patient develops symptoms not suggestive of dengue, looking for other causes is necessary.

More than 80% of children in our study were classified as having mild COVID-19 as well as non-severe dengue (dengue without/with warning signs), with nearly two-thirds of cases in this category. In general, the majority of children with SARS-CoV-2 infection are asymptomatic or mild, and the majority of dengue cases in children are also non-severe [15,16]. This trend was also observed in pediatric coinfection in a study with a predominance of mild COVID-19 and dengue with warning signs; no asymptomatic cases were identified in the coinfection [7]. Clinical characteristics between the severe and non-severe groups in our study showed almost no statistically significant difference. A study from the Philippines showed that there was no difference in outcomes between the coinfection group and the SARS-CoV-2 monoinfection group [7]. In our study, no patients died, while literature reported that the mortality rates of dengue infection, COVID-19, and the coinfection were less than one percent, 0.3%, and 16.13-28% (in adults), respectively [3,6,8,17]. Therefore, we hypothesize that children with coinfection have a better prognosis than adults. However, further studies with larger sample sizes are needed to confirm this hypothesis.

Thrombocytopenia was common, with more than 80% of cases exhibiting this condition. In dengue, thrombocytopenia, leukopenia, and increased hematocrit are characteristic manifestations that help diagnose and predict the severity of plasma leakage and organ damage [18,19]. In COVID-19, mild thrombocytopenia (less than 150x10^3^/µL) is also commonly observed in 70-95% of severe patients [20]. Literature shows conflicting results regarding the prognostic potential of thrombocytopenia in COVID-19 [21-24]. Our study recorded a platelet count below 70x10^3^/µL, and in the case with DSS, the platelet count was much lower. This seems to be due to the impact of DENV infection. Therefore, if a child with COVID-19 experiences a significant decrease in platelet count, the possibility of coinfection with another agent should be considered. Serum ferritin is a predictor of severe dengue and COVID-19 [25,26]. Our study showed elevated serum ferritin in all cases, consistent with the results of other studies performed in adults [3,5].

It is difficult for clinicians to diagnose and choose the most appropriate treatment in children with coinfection. The use of any treatment can be a “double-edged sword”. Anticoagulants are recommended for severe/critical COVID-19 patients to prevent thrombotic complications. However, anticoagulants can aggravate bleeding, commonly seen in DENV infection. The same applies to fluid therapy in dengue treatment, which can worsen respiratory failure due to COVID-19; the antiviral drug (remdesivir) can worsen liver damage due to dengue infection [3]. As a result, patients with the coinfection need to be closely monitored for clinical symptoms and laboratory parameters to determine the patient's main condition (COVID-19 or dengue infection or both), and then decide whether to treat dengue or COVID-19 or change treatment appropriately (reduction of anticoagulant dose, coagulation test, respiratory support with CPAP, mechanical ventilation if the patient has respiratory failure) [9,10].

An underlying condition is a crucial feature in both dengue and COVID-19 because it is related to the outcome of the diseases. Children with comorbidities (neurologic diseases, cardiovascular diseases, obesity, immunodeficiency) may be at higher risk of severe COVID-19 [11]. Meanwhile, some epidemiological studies have recognized several risk factors for severe dengue, including malnutrition, obesity, diabetes, hypertension, and chronic allergies [27]. In the coinfection, many studies have shown that the presence of comorbidities increased the risk of severe illness and death [3,5]. In our study, obesity was the most common comorbidity, accounting for approximately three-quarters of cases with comorbidities. This condition is one of the most common comorbidities and is a risk factor for severe disease in both COVID-19 and dengue [27,28]. Our study results, together with evidence in the literature, indicate a potentially high risk for obese children with coinfection and an urgent need to proceed with prevention efforts such as adequate vaccination and appropriate care.

Coinfected patients with abdominal pain and petechiae need to be monitored more carefully because our study identified these symptoms as independent factors associated with severe disease with ORs of 11.25 and 10.667, respectively. Another study in Vietnam shows that abdominal pain, spontaneous petechiae, vomiting, and headache were related to severe dengue with complications [29]. Approximately two-thirds of patients with DSS had bleeding. Bleeding complications were more common in dengue cases with shock [29]. Abdominal pain, petechiae, and mucosal bleeding are specific symptoms suggesting dengue infection; therefore, we hypothesize that severe cases of the coinfection were mainly caused by dengue [27].

This study has several limitations. First, in the inclusion criteria, the diagnosis of dengue was made when there was either a positive NS1Ag or a positive Dengue IgM ELISA. However, the diagnosis of COVID-19 and dengue coinfection must rely on RT-PCR for both viruses. Many studies have shown that there may be cross-reactivity of the Dengue IgM/IgG ELISA test with antibodies formed after SARS-CoV-2 infection [30]. Second, coinfection with other agents, such as bacteria and viruses, has not been fully evaluated. Third, the study's sample size was small and based on only one hospital, lacking an assessment of outpatient patients with coinfection, as well as COVID-19 cases that had not been tested for dengue infection. Fourth, the inherent limitations of the retrospective study include potential biases and the inability to establish causality. Therefore, in further studies, other potential confounding variables, such as the predominant SARS-CoV-2 variants, the ability to access initial healthcare, and socioeconomic status, should be considered in detail to explain whether these factors could influence the severity of the disease. To our knowledge, this is the first study that describes characteristics of the coinfection in the pediatric population in Vietnam, so future multicenter studies on a larger population are required.

## Conclusions

SARS-CoV-2 and dengue coinfection in children remains a health concern in tropical and subtropical countries of the Asia-Pacific and Latin American regions, including Vietnam. Although most children have mild COVID-19 and disease progression similar to patients with dengue infection alone, some children may have severe COVID-19 and dengue coinfection. This study has highlighted some clinical and laboratory characteristics of coinfection in children and the need for guidelines on the diagnosis and management of the coinfection. Comorbidities, especially obesity, and some clinical symptoms, including abdominal pain and petechiae, were identified as independent risk factors for severe illness. Further studies with multicenters and a larger sample size are needed to assess and confirm the hypotheses of the coinfection more thoroughly.

## Appendices

Appendix 1 

**Table 5:** Severity classification based on dengue hemorrhagic fever guideline of the Ministry of Health (Vietnam). AST: Aspartate Aminotransferase; ALT: Alanine Aminotransferase.

Severity	Dengue without warning signs	Dengue with warning signs	Severe dengue
Features	Living/traveling to an epidemic region. Fever ≤ 7 days and 2 of the following signs:	At least 1 of the following signs:	At least 1 of the following signs:
	- Nausea, vomiting	- Struggling, lethargy	1. Severe plasma leakage:
	- Rash	- Severe abdominal pain or severe pain in the liver area.	- Dengue shock syndrome
	- Myalgia, arthalgia, retroorbital pain	- Severe vomiting ≥ 3 times/1 hour or ≥ 4 times/6 hours.	- Fluid overload, signs of respiratory failure.
	- Petechiae	- Mucosal bleeding:	2. Severe bleeding
	- Hematocrit is normal or increased	- Hepatomegaly > 2cm below the costal margin.	3. Organ failure
	- White blood cells are normal or decreased	- Oliguria.	- Liver: AST or ALT ≥ 1000U/L.
	- Platelets are normal or decreased.	- Hematocrit increases with rapid decrease in platelet count.	- Central nervous system: disturbance of consciousness.
		- AST/ALT ≥ 400U/L.	
		- Pleural or peritoneal effusion on ultrasound or X-ray.	

Appendix 2 

**Table 6:** Severity classification based on pediatric COVID-19 guidelines of the Ministry of Health (Vietnam).

Mild	Moderate	Severe	Critical
- No symptoms and signs OR	- Tachypnea for age	- Severe pneumonia: Tachypnea and chest indrawing or grunting (< 2 months old); nasal flaring.	- Mechanical ventilation
- Atypical signs: fever, sore throat, cough, runny nose, diarrhea, vomiting, myalgia, loss of tastr/smell.	- SpO2: 94-95% in room air.	- SpO2: 90-< 94% in room air.	- Cyanosis or SpO2 <90% in room air.
- SpO2 ≥ 96% in room air.	- Awake, can eat and drink	- Tired of breastfeeding, eating poorly	- Shock
- Normal respiratory rate for age.	- Interstitial lesions/the ground glass on chest X-rays.	- Lung lesions ≥ 50% on chest X-rays	- Multiorgan failure.
- Awake			- Cytokine storm.

## References

[1] Guzman MG, Gubler DJ, Izquierdo A, Martinez E, Halstead SB (2016). Dengue infection. Nat Rev Dis Primers.

[2] (2024). Comprehensive Guideline for Prevention and Control of Dengue and Dengue Haemorrhagic Fever. Revised and expanded edition. https://iris.who.int/handle/10665/204894.

[3] Prapty CN, Rahmat R, Araf Y (2023). SARS-CoV-2 and dengue virus co-infection: Epidemiology, pathogenesis, diagnosis, treatment, and management. Rev Med Virol.

[4] (2024). Hướng dẫn chẩn đoán, điều trị sốt xuất huyết Dengue (Ban hành kèm theo quyết định số 3705/QĐ-BYT ngày 22/8/2019). https://thuvienphapluat.vn/van-ban/The-thao-Y-te/Quyet-dinh-3705-QD-BYT-2019-Huong-dan-chan-doan-dieu-tri-sot-xuat-huyet-Dengue-422657.aspx.

[5] Tsheten T, Clements AC, Gray DJ, Adhikary RK, Wangdi K (2021). Clinical features and outcomes of COVID-19 and dengue co-infection: a systematic review. BMC Infect Dis.

[6] Badal S, Thapa Bajgain K, Badal S, Thapa R, Bajgain BB, Santana MJ (2021). Prevalence, clinical characteristics, and outcomes of pediatric COVID-19: a systematic review and meta-analysis. J Clin Virol.

[7] Pantig FM, Clemens SA, Clemens R, Maramba-Lazarte CC, Madrid MA (2023). SARS-CoV-2 and dengue coinfection in Filipino children: epidemiology profile, clinical presentation and outcomes. Pediatr Infect Dis J.

[8] Mejía-Parra JL, Aguilar-Martinez S, Fernández-Mogollón JL, Luna C, Bonilla-Aldana DK, Rodriguez-Morales AJ, Díaz-Vélez C (2021). Characteristics of patients coinfected with severe acute respiratory syndrome coronavirus 2 and dengue virus, Lambayeque, Peru, May-August 2020: a retrospective analysis. Travel Med Infect Dis.

[9] (2024). Hướng dẫn chẩn đoán và điều trị COVID-19 ở trẻ em (Ban hành kèm theo quyết định số 5155/QĐ-BYT ngày 8/11/2021). https://thuvienphapluat.vn/van-ban/The-thao-Y-te/Quyet-dinh-5155-QD-BYT-2021-huong-dan-chan-doan-va-dieu-tri-COVID19-o-tre-em-493731.aspx.

[10] (2024). Hướng dẫn chẩn đoán và điều trị COVID-19 ở trẻ em (Ban hành kèm theo quyết định số 2959/QĐ-BYT ngày 24/07/2023). https://thuvienphapluat.vn/van-ban/The-thao-Y-te/Quyet-dinh-2959-QD-BYT-2023-Huong-dan-chan-doan-va-dieu-tri-COVID19-o-tre-em-573808.aspx.

[11] Nguyen PN, Thuc TT, Hung NT (2022). Risk factors for disease severity and mortality of children with Covid-19: a study at a Vietnamese Children's hospital. J Infect Chemother.

[12] Rana MS, Alam MM, Ikram A, Zaidi SSZ, Salman M, Khurshid A (2024). Bản tin phòng chống dịch COVID-19 ngày 11/4 của Bộ Y tế. Journal of Medical Virology.

[13] Temsah MH, Barry M, Memish ZA, Al-Tawfiq JA (2023). Celebrating the 2023 New Year at the time of the tridemic shadow. New Microbes New Infect.

[14] Harapan H, Ryan M, Yohan B (2021). Covid-19 and dengue: double punches for dengue-endemic countries in Asia. Rev Med Virol.

[15] Irfan O, Muttalib F, Tang K, Jiang L, Lassi ZS, Bhutta Z (2021). Clinical characteristics, treatment and outcomes of paediatric COVID-19: a systematic review and meta-analysis. Arch Dis Child.

[16] Idrus NL, Md Jamal S, Abu Bakar A, Embong H, Ahmad NS (2023). Comparison of clinical and laboratory characteristics between severe and non-severe dengue in paediatrics. PLoS Negl Trop Dis.

[17] Lam PK, Tam DT, Diet TV (2013). Clinical characteristics of dengue shock syndrome in Vietnamese children: a 10-year prospective study in a single hospital. Clin Infect Dis.

[18] Tsheten T, Clements AC, Gray DJ, Adhikary RK, Furuya-Kanamori L, Wangdi K (2021). Clinical predictors of severe dengue: a systematic review and meta-analysis. Infect Dis Poverty.

[19] Thach TQ, Eisa HG, Hmeda AB (2021). Predictive markers for the early prognosis of dengue severity: a systematic review and meta-analysis. PLoS Negl Trop Dis.

[20] Levi M, Thachil J, Iba T, Levy JH (2020). Coagulation abnormalities and thrombosis in patients with COVID-19. Lancet Haematol.

[21] Choi JH, Choi SH, Yun KW (2022). Risk factors for severe COVID-19 in children: a systematic review and meta-analysis. J Korean Med Sci.

[22] Huang C, Wang Y, Li X (2020). Clinical features of patients infected with 2019 novel coronavirus in Wuhan, China. Lancet.

[23] Lippi G, Plebani M, Henry BM (2020). Thrombocytopenia is associated with severe coronavirus disease 2019 (COVID-19) infections: a meta-analysis. Clin Chim Acta.

[24] Zong X, Gu Y, Yu H, Li Z, Wang Y (2021). Thrombocytopenia is associated with COVID-19 severity and outcome: an updated meta-analysis of 5637 patients with multiple outcomes. Lab Med.

[25] Soundravally R, Agieshkumar B, Daisy M, Sherin J, Cleetus CC (2015). Ferritin levels predict severe dengue. Infection.

[26] Kaushal K, Kaur H, Sarma P (2022). Serum ferritin as a predictive biomarker in COVID-19. A systematic review, meta-analysis and meta-regression analysis. J Crit Care.

[27] Zulkipli MS, Dahlui M, Jamil N, Peramalah D, Wai HV, Bulgiba A, Rampal S (2018). The association between obesity and dengue severity among pediatric patients: a systematic review and meta-analysis. PLoS Negl Trop Dis.

[28] Woodruff RC, Campbell AP, Taylor CA (2022). Risk factors for severe COVID-19 in children. Pediatrics.

[29] Phuong CX, Nhan NT, Kneen R (2004). Clinical diagnosis and assessment of severity of confirmed dengue infections in Vietnamese children: is the world health organization classification system helpful?. Am J Trop Med Hyg.

[30] Lustig Y, Keler S, Kolodny R (2021). Potential antigenic cross-reactivity between severe acute respiratory syndrome coronavirus 2 (SARS-CoV-2) and dengue viruses. Clin Infect Dis.

